# The role of red blood cell exchange in sickle cell disease in patient with COVID‐19 infection and pulmonary infiltrates

**DOI:** 10.1002/ccr3.3526

**Published:** 2020-11-16

**Authors:** Lina Okar, Maya Aldeeb, Mohamed A. Yassin

**Affiliations:** ^1^ Department of Medical Education Hamad Medical Corporation Doha Qatar; ^2^ Department of Medical Oncology, Hematology Section National Center for Cancer Care & Research Hamad Medical Corporation Doha Qatar

**Keywords:** acute chest syndrome, COVID‐19, hemoglobinopathies, red blood cell exchange, Sickle cell disease

## Abstract

Due to the overlap between ACS and COVID‐19 pneumonia, we recommend close monitoring for those patients and offering them RBC exchange early in the course of the disease to avoid clinical deterioration.

## Graphical abstract

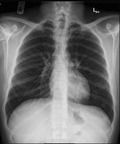

## 1 INTRODUCTION

COVID‐19–related pneumonia overlaps with ACS, one of the most common acute presentations in SCD patients. We present a case of 22‐year‐old SCD patient presented to the emergency department with mild respiratory symptoms turned out to be COVID‐19 positive and received red blood cell exchange to avoid the possibility of deterioration.

COVID‐19 also known as severe acute respiratory syndrome coronavirus 2 (SARS‐CoV‐2) is the novel virus that caused the latest pandemic in the world. The most serious presentation of this virus was acute respiratory distress syndrome (ARDS) which has high mortality rate. Elderly patient and those with multiple comorbidities have been categorized as high‐risk group for developing serious complication from COVID‐19 infection.^1^

SCD is one of the most common hemoglobinopathies worldwide, and it affects the shape of red blood cells (RBCs) which leads to several clinical manifestations. Complications might be acute or chronic, and the most important and common acute complications are vaso‐occlusive crisis (VOC) and acute chest syndrome (ACS).^2^ Respiratory infections are considered an important trigger for ACS.^3^ The impact of COVID‐19 infection in SCD patients is still not clear, and data are conflicting. However, some experiences suggested low mortality and morbidity rate in SCD patients with COVID‐19 infection.^4^ Previously published researches mentioned the overlap between COVID‐19 pneumonia and ACS^1^ which we believed rises a challenge for physician, and thus, we recommend that those patients should be monitored closely as deterioration in their clinical status was prescribed in multiple case reports.^5^ The definitive management plan in this situation is not defined clearly till now. Whether applying early red blood cell exchange can prevent further deterioration is still a question that needs to be answered.

## 2 CASE PRESENTATION

A 22‐year‐old man presented to the hospital with history of fever, sore throat, mild nonproductive cough, generalized body ache, chest pain, fatigue, and decreased appetite for three days. The patient has history of sick contact history with an a positive COVID‐19 case, no recent travel. His past medical history was remarkable for non‐transfusion dependent Sickle cell disease taking Hydroxyurea 500 mg daily, no previous surgeries, previous recurrent painful crisis most of them did not require hospital admission and presented as generalized pain, pain in the right arm and left hip which appeared to be avascular necrosis, last painful crisis was 7 months before as lower limb pain for which he was given only tramadol. Primary investigations including laboratory tests, nasopharyngeal swab for COVID‐19 PCR, and chest X‐ray were performed, and the results are shown in Table 1.

**Table 1:** Investigations

Investigations	
Chest X‐ray: Figure 1.	Small ill‐defined patchy opacity is seen at right lung lower zone periphery, differential diagnosis includes viral infection. Clear both costophrenic angles. Normal cardio‐thoracic ratio.
ECG	Corrected QT: 379 ms, no signs of ischemic change, normal QRS and StT segment.
Blood tests:	
ABO	Group B +
Hemoglobin (13‐17 gm/dL)	12.8
RBCs (4.5‐5.5 x 10^6^)	4.0
WBCs (4‐10 X 10^3^/UL)	3.2
ANC (2.0‐7.0 × 10^3^/UL)	1.76
PLT (150‐400 × 10^3^/UL)	77
Reticulocyte (0.2%‐2.5%)	1.1
Lymphocytes count (1‐3 x 10^3^/UL)	1.03
Ferritin (8‐252 mcg/L)	608 (comparing with 49 last value)
IL6 ( 6 −9 pg/mL)	9
CRP (0‐5 Umg/L)	9.5 on admission 2 on discharge
D‐dimer (0.00 −0.4 mcg/L)	0.41
LDH (135‐214 U/L)	199
Renal function tests (Urea/ Cr) (2.1‐ 8.8mmol/L) (44‐ 80Umoll/L)	Ur 2 Cr 67
ALT/ AST ( 0‐33 U/L) ( 0‐32 U/L)	12.6 20
TB (0‐21 umol/L_ DB (0‐5 Umol/L)	43 11
Albumin (35‐50 gm/L)	46
G6PD	Normal
Hemoglobin electrophoresis	Hgb A 0.0 Hgb A2 3.0 Hgb F 26.9 Hgb S 70.1
COVID‐19 PCR	Positive
Outcome	Favorable

**Figure 1:**
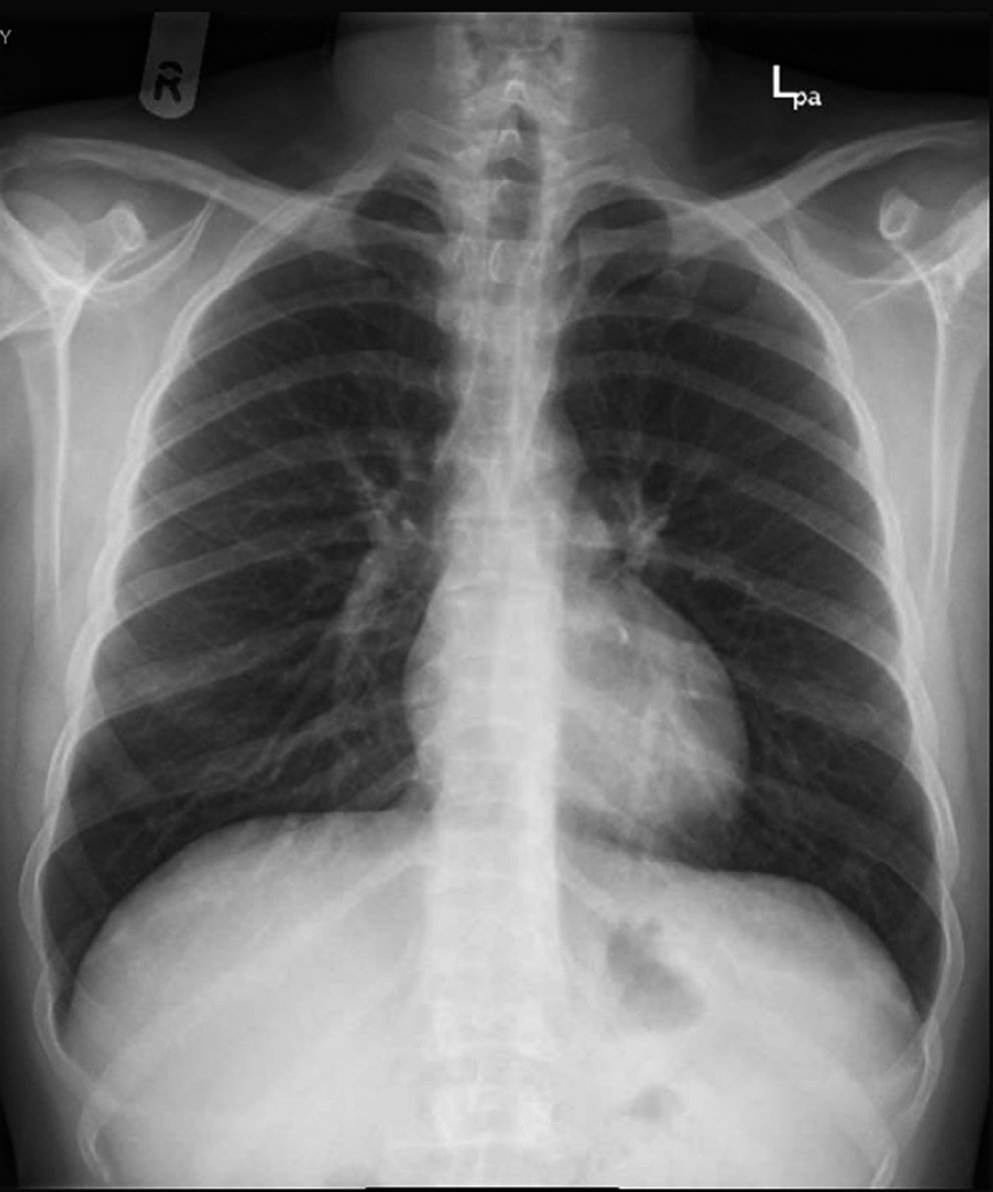
Chest X‐ray

To this point, the patient differential diagnosis was as follows: Acute chest syndrome triggered with COVDI‐19 infection or viral pneumonitis. He was admitted to the intensive care unit (ICU) after considering him as high risk for COVID‐19 complication. The decision was made to do red blood cell exchange early in the course of the infection to avoid possible deterioration in his case and the need of intubation. Upon admission to the ICU, his vital signs were as follows: temperature 36.4, heart rate 69/min, respiratory rate 20/ min, blood pressure 117/59 mm Hg, and oxygen saturation of 96% on room air, and he did not require any oxygen supplementation in ICU. He was on the following medications: Lopinavir/ Ritonavir (Kaletra) 200/50 mg for two days which later stopped and cefuroxime 1.5 g daily was given for 7 days, Hydroxyurea 500 mg daily, and enoxaparin 40 mg SC as thrombosis prophylaxis, although he had thrombocytopenia the benefit from offering him thrombosis prophylaxis outweigh the risk of bleeding. Plasma exchange with 6 units of PRBCs was done on the second day of hospital admission without any complications. He stayed in the ICU for 4 days for observation and then transferred to the ward; during ICU admission, no deterioration was happened, and in the ward, repeated chest X‐ray was normal. After 6 days in the ward without any deterioration in his clinical course with resolving respiratory symptoms, the patient was discharged to a quarantine facility (Figure 1).

## 3 DISCUSSION

Coronavirus is the novel virus responsible for the latest pandemic declared by World Health Organization (WHO) on March 11, 2019. Taking a good history to define the risk of infection is critical as patient might be asymptomatic. However, symptomatic patients may present with fever, dyspnea, Che, fatigue, and generalized muscle ache. Other non‐typical symptoms like gastrointestinal were also reported.^6^ Severity of the disease differs among affected patients for example elderly, and patients with comorbidities like hypertension, diabetes, and cardiovascular diseases are at more risk for complication and worse clinical course than normal population.^7^

Sickle cell disease (SCD) is an inherited hemoglobinopathy with main characteristic being the presence of hemoglobin S (HbS). The inheritance might be in homozygous or heterozygous form with a disease severity that differs accordingly. This hemoglobin causes deformity in the structure of red blood cells (RBCs) changing them to sickle‐shaped, rigid, and dysfunctional RBCs. Clinical manifestations are vaso‐occlusive crisis (VOC), intra‐ and extra‐vascular hemolytic anemia.^2^ Complication of SCD can be categorized into acute and chronic.

Acute complications include acute chest syndrome (ACS), vaso‐occlusive crisis (VOC), hepatobiliary complications, stroke, splenic sequestration, priapism, acute anemia, and fever.^8^ Chronic complications are pulmonary hypertension, hepatic iron overload, kidney disease, avascular necrosis, retinopathy, and legs ulcer.^7^, ^9^, ^10^, ^11^, ^12^ Among the acute complications, the most common are VOC and ACS. VOCs are episodes of severe pain due to microvascular occlusion with erythrocytes and leukocytes, thus preventing blood flow and causing organ ischemia. ACS is common lung insult in SCD patients, known as newly pulmonary infiltrate due to alveolar consolidation affecting one lung segment at least, in it is severe form it is similar to the acute respiratory distress syndrome (ARDS), along with the radiological findings, patients usually present with fever, chest pain, shortness of breath, cough and wheezing. It is considered the second most common cause of hospitalization and the main reason behind intensive care unit admission as well as early death among those patients.^3^ There are three previously mentioned causes of ACS: first, pulmonary infection; second, bone marrow fat embolization; and third, intra‐vascular pulmonary sequestration. Among those causes, pulmonary infections are the most common one and it is usually due to community‐acquired pathogen that causes over‐inflammatory response instead of mild upper respiratory picture.^3^ The National Acute Chest Syndrome Study published by Vichinsky et al to define the causes of ACS showed that infections are the main cause with atypical bacteria and viruses being the major causes, and despite the splenic dysfunction in SCD, encapsulated bacteria were rarely isolated.^13^

Patients with hemoglobinopathies are considered high risk for developing severe complication from COVID‐19 infection as per the Thalassemia International Federation.^14^ However, no strong evidence is available in this regard, and it is not well known if COVID‐19 infection really increase the morbidity and mortality in SCD patients or not.^4^ The overlap between the ACS and COVID‐19 pneumonia has been described.^15^ Thus, taking a final decision in this regard is hard because of the conflict in literature and variance of COVID‐19 clinical course among SCD population, and we summarized all previously published data in Table 2.

**Table 2:** Literature review

Case series and Case reports:											
Reference	Patient Number	Age	gender	Past medical history	Presenting symptoms	Clinical course	Radiology findings (chest X‐ray or CT)	Management	ICU Admission	Length of stay	Outcome
1	4	32	Male	Recurrent VOC ACS Lower limb ulcer	VOC	Severe	Positive	Ceftriaxone Azithromycin HDQ **Blood transfusion**	Yes	13 days	Favorable
		22	Female	Recurrent VOC ACS Asthma	VOC GI symptoms	Mild	Negative	Ceftriaxone Pain medication	No	2 days	Favorable
		37	Female	Recurrent VOC ACS Venous thrombosis	VOC	Mild	Negative	Pain medications	No	8 days	Favorable
		41	Male	Avascular necrosis (bilateral hip) Pulmonary embolism	Respiratory symptoms Hip pain (VOC)	Mild	Not available	Pain medications	No	4 days	Discharged against medical advice
16	2	24	Male	Minor VOC	Chest pain (ACS)	Moderate	Positive	Amoxicillin‐clavulanate	No	3 days	Favorable
		20	Female	Recurrent VOC	VOC Low oxygen saturation	Moderate	Negative	Pain management	No	NA	Favorable
5	1	21	Male	Recurrent VOC ACS Avascular necrosis of the hip	VOC Fever	Mild	positive	Ceftriaxone, azithromycin HCQ **Blood transfusion Exchange transfusion**	NA	16 days	Favorable
17	1	45	male	Sickle cell nephropathy Ischemic Retinopathy Priapism Cardiac remodeling ACS	multifocal VOC and fever	severe	positive	Amoxicillin‐clavulanic acid HCQ O2 supplementation Tocilizumab and **Blood transfusion**	No	5 days	Favorable
15	1	27	Male	VOCs	VOC Fever Respiratory symptoms	severe	Positive	Ceftriaxone Doxycycline Piperacillin/ tazobactam HCQ Tocilizumab methylprednisolone O2 supplementation **Exchange transfusion**	Yes	12 days	Favorable
18	1	35	Female (Pregnant)	ACSs Pulmonary thromboembolism Pulmonary Hypertension Leg ulcers preeclampsia in a previous pregnancy	Fever Myalgia Respiratory symptoms Low Oxygen saturation	Severe	positive	O2 supplementation Ceftriaxone Azithromycin **Blood transfusion**	Yes	9 days	Favorable
19	1	18	Female	VOCs Acute intrahepatic cholestasis	VOC	severe	Positive	Azithromycin Pain medications **Simple transfusion**	Yes	16 days	Favorable
20	3	23	Female	VOCs	Fever Cough Abdominal pain weight loss	Mild	Positive	Clarithromycin **Exchange transfusion**	No	2 days	Favorable
		44	Male	VOCs hypertension Renal insufficiency	symptomatic leg ulcer coughing and slight dyspnea	Initially mild deteriorated	Positive	HCQ **Exchange transfusion**	NO	30 days	Favorable
		23	Female	NA	VOC AKI Vomiting	Moderate complicated with DVT	Positive	Ceftriaxone Metronidazole Oxygen supplementation Enoxaparin **Exchange transfusion**	NO	51 days	Favorable
21	3	14	Female	VOCs	VOC	Mild	Negative	Pain medications ceftriaxone	No	10 days	Favorable
		12	Male	Splenectomy	VOC	Mild initially then severe	Negative Initially Repeated positive	Ceftriaxone Azithromycin Oxygen supplementation **blood transfusion** dexamethasone HCQ	No	12 days	Favorable
		50	Female		Asymptomatic	Asymptomatic	Negative	none	none	none	Favorable

Other larger studies:											
Reference	Patient Number	Age	gender	Past medical history	Presenting symptoms	Clinical course	Radiology findings (chest X‐ray or CT)	Management	ICU Admission	Length of stay	Outcome
22	83	30 (0.3‐68)	38 Male 45 Female	48/83 ACS 44/83 VOC	44 VOC 23 ACS	NA	NA	**6 simple transfusion 7 exchange transfusion**	17 Admitted 9 intubated 2 ECMO	8 (2‐37)	2 died This study suggests the following: ‐COVID‐19 does not seem to increase the morbidity or mortality in patients with SCD. ‐ VOC can complicate COVID‐19 infection. ‐ Patients with SCD should be monitored closely during hospitalization as they might deteriorate easily.
23	10	range, 23‐57)		5 of them recurrent VOC One CKD One stroke Two: iron overload	Fever Dry cough Hypoxemia 8/10 presented with VOC	9 mild 1 severe	5/10 positive	8 of them enoxaparin **3 needed blood transfusion** One severely ill, only palliative treatment One needed peritoneal dialysis	none	Mean length of stay 7.2 days (range, 3‐17)	9 Favorable 1 death
24	24 patients	9 > 60 15 < 60	6 Male 18 Female	12 HTN 12 obesity 9 DM 3 asthma 3 ESRD 5 malignancy 6 VTE 1 COPD	Fever 79% Cough83%Myalgia 67% VOCs 4	NA	NA	**4 blood transfusion** HCQ and steroid for all patient 7 required o2 supplementation	1 ICU admission	Mean 7.6 days SCT IN SCD	1 death ‐Patient with sickle cell trait had mild course of disease with lower change of complications but longer hospital stays.
25	178 12 hospitalized	<19 44 >19 134	101 Females 76 Males	96 recurrent pain crises 57 > 1‐episode ACS 23 chronic transfusion 23 PAH 32 Stroke 56 renal disease		11 asymptomatic 96 mild 32 moderates 30 severe 9 critical	NA	**68 blood** **transfusion** **16 Exchange transfusion** 4 required dialysis	19 10 needed intubations		13 death ‐SCD can cause multisystem organ damage, life‐long disability, and reduced lifespan. ‐SCD is one of many possible explanations for higher rates of illness and mortality from COVID‐19 among Black populations in the United States
4	9	Mean 27.9	5 Male 4 Female				8/9 positive findings	**6 received blood transfusion**	1 Intubation 0	0‐16 days	

As we notice from the table above, few published cases described the use of RBC exchange to manage ACS in SCD patients. Whenever RBCs exchange was used it was because those patients deteriorated, interestingly all of them improved after the exchange that is why we should raise the following question “ is it necessary to leave this choice as a rescue option?”. Previously similar published case described the role of early RBCs exchange in preventing further deterioration in SCD patient, in that case they offer RBCs exchange once oxygen requirement started to increase.^15^ The difference in our case is that we chose to offer our patient RBC exchange even when he was stable due to the overlapping between ACS and COVID‐19 pneumonia, and by weighing the benefit and risk, we believe that giving him RBC exchange played an important role in alleviating the infection course as well as the need of ICU admission and possible intubation.

Both pneumonia and acute chest syndrome are life‐threatening conditions. Red blood cell exchange is a well‐known method to treat ACS, and there are published data about using it in case of severe COVID‐19 pneumonia, mostly after deterioration.^1^, ^5^, ^15^ Here, we present the first case report for SCD patient infected with COVID‐19 who received red blood cell exchange immediately after admission to avoid the deterioration and need of intubation giving him the benefit of doubt. We think offering RBC exchange for patients with SCD and COVID‐19 pneumonia upon diagnosis may have major benefits such as avoiding ICU admission and intubation.

## 4 CONCLUSION

With the latest pandemic due to COVID‐19 infection, a lot of patients were vulnerable and at risk of developing severe and fatal complications. Patients with SCD suffer from multiple acute and chronic complications. The exact clinical course of COVID‐19 infection in those patients is not yet final, and conflicting data are available. However, delaying appropriate management may carry an increased risk for intubation and mortality. We recommend that physicians should keep a low threshold for admitting SCD patients in whom they suspect COVID‐19 infection and to monitor them closely as well provide RBCs exchange initially in the disease course to give them the benefit of doubt. More research is needed to reach a high evidence regarding the management plan.

## CONFLICT OF INTEREST

None declared.

## AUTHOR CONTRIBUTIONS

All authors contributed equally in writing the manuscript.

## STATEMENT OF ETHICS

Consent was obtained from the patients. Case was approved by HMC Medical Research Center.

## Data Availability

All data related to this article are available upon request.
